# Characterization of global research trends and prospects on celastrol, a principal bioactive ingredient of *Tripterygium wilfordii* Hook F: bibliometric analysis

**DOI:** 10.1080/13880209.2024.2443424

**Published:** 2025-01-02

**Authors:** Huizi Ye, Yufang Wang, Xue Zhang, Lin Yang, Banglan Cai, Denghai Zhang, Bin Peng

**Affiliations:** a Postgraduate training base at Shanghai Gongli Hospital, Ningxia Medical University, Shanghai, China; b Shanghai Health Commission Key Lab of Artificial Intelligence (AI)-Based Management of Inflammation and Chronic Diseases, Department of Central Laboratory, Gongli Hospital of Shanghai Pudong New Area, Shanghai, China; c School of Basic Medicine, Ningxia Medical University, Yinchuan, Ningxia, China

**Keywords:** Celastrol, bibliometrics, visualization, CiteSpace, VOSviewer

## Abstract

**Context:**

Celastrol, acknowledged as a prominent exemplar of the potential for transforming traditional medicinal compounds into contemporary pharmaceuticals, has garnered considerable attention owing to its extensive pharmacological activities. The increasing volume of publications concerning celastrol highlights its importance in current scientific inquiry. Despite the growing interest in this compound, a bibliometric analysis focused on this subject remains to be undertaken.

**Objective:**

Our study explored a bibliometric approach to identify and characterize global research trends and frontiers related to celastrol, including mapping research outputs, influential contributors, and thematic areas, as well as highlighting gaps and opportunities for future investigations.

**Materials and methods:**

In this study, we utilized the Web of Science Core Collection (WoSCC) to source and review articles related to celastrol published from 1997 to 2023. The bibliometric analysis was conducted using the R package ‘Bibliometrix,’ supplemented by visualization tools including CiteSpace, VOSviewer, and GraphPad Prism 10.

**Results:**

Celastrol related research papers have exhibited an upward trend annually and can be categorized into three distinct phases, each highlighting different areas of focus. China, the United States, and South Korea rank as the top three nations for publication volume, with varied research interests across these countries. Several prolific research teams have emerged, each with distinct areas of interest. Examining the primary research domains of celastrol (anti-inflammatory, anticancer, and toxicity) reveals a notable intersection between the first two domains.

**Discussion and Conclusions:**

The scope and depth of celastrol research have been steadily expanding, with regional and team-specific variations. Key research areas include anti-inflammatory, anticancer, and toxicity studies. Future research is expected to focus on enhancing the effectiveness and reducing the toxicity of celastrol. Meanwhile, given the multi-target characteristics of celastrol’s effects, integrating methods such as network biology and molecular simulation will provide a novel perspective for celastrol research.

## Introduction

*Tripterygium wilfordii* Hook F (TwHF), belonging to the Celastraceae family of vine-like plants, holds a pivotal place in traditional Chinese medicine, with its first publication in the 16th-century seminal work, the *Compendium of Materia Medica*. In contemporary medicine, TwHF has been recognized for its efficacy in treating autoimmune disorders, notably rheumatoid arthritis and systemic psoriasis (Liu et al. 2013; Wu et al. 2015). Celastrol, a principal bioactive constituent derived from TwHF, exhibits a spectrum of pharmacological effects (Guo et al. 2005). These include antitumor, antioxidant, anti-obesity, neuroprotective and immunosuppressive effects (Liu et al. 2015; Hou et al. 2020; Xu et al. 2021; Zhao et al. 2023), underscoring its potential therapeutic applications across a diverse range of conditions.

Natural products of medicinal plants are an essential source for the screening and development of new drugs (Patridge et al. 2016), which are highly targeted, have fewer side effects, and are less resistant than traditional chemotherapeutic drugs (Deng et al. 2020). As early as in 2006, research identified celastrol as a natural proteasome inhibitor, highlighting its significant potential in cancer prevention and therapy (Yang et al. 2006). Following this discovery, celastrol was recognized by *Cell* in 2007 as one of the top five natural products with promising drug development potential (Corson and Crews 2007), the anti-inflammatory and antioxidant is the core bioactivity of TwHF (Li and Hao 2019; Luo et al. 2022), and it possesses a wide range of pharmacological activities. With its multi-target and multi-mechanism characteristics, celastrol exemplifies the transformation of traditional Chinese herbal medicine into modern pharmaceuticals, offering extensive developmental value and application prospects.

Despite the promising pharmacological profile of celastrol, its clinical application faces significant challenges due to poor solubility and potential toxic reactions. These limitations have become a substantial bottleneck, impeding the widespread adoption of celastrol in clinical practice. Consequently, it becomes critically important to conduct a more comprehensive analysis of the existing research on celastrol to overcome these barriers. In light of these considerations, the following bibliometric analysis is proposed to guide and inform future research endeavors on celastrol.

Bibliometrics constitutes an interdisciplinary domain that applies quantitative analyses to all forms of knowledge carriers through mathematical and statistical methodologies (Smith 2008). This analytical approach is adept at pinpointing significant nodes and distilling valuable insights from extensive datasets (Moed et al. 1995), with a primary focus on the subjective interpretation of content, supplemented by the utilization of scientometric tools for objective analysis. Employed extensively in the enhancement of literature review studies, bibliometrics serves the crucial function of examining the core attributes of previously published works on a given topic. Concurrently, research that employs a knowledge mapping approach for quantitative analysis can more objectively delineate the research hotspots and trends within a specific domain (Ma et al. 2020), offering a comprehensive view of the field’s evolution and current focal points.

In this study, we conducted a systematic bibliometric analysis to assess the landscape of celastrol research spanning from 1997 to 2023. Leveraging the capabilities of CiteSpace (Chen 2006), VOSviewer (van Eck and Waltman 2010), and Bibliometrix (Aria and Cuccurullo 2017), we embarked on a comprehensive examination of various dimensions including publication output, disciplinary diversity, geographical distribution of contributing countries/regions, journals, categories diversity, authors, core teams, keywords and topics. The findings of our analysis culminated in the creation of a visualization map that delineates the global research landscape of celastrol. The visual representation is intended to aid researchers, by enhancing their comprehension of the prevailing research paradigms and guiding them towards future investigative pathways.

## Materials and methods

### Database selection and search strategy

Data were sourced from the Science Citation Index Expanded (SCI-E) within the Web of Science (WoS) Core Collection database (Mongeon and Paul-Hus 2016). The search query was TS = (celastrol OR tripterine), covering the period from January 1, 1997, to December 31, 2023. This yielded a total of 1,426 publications on celastrol. Publications in English were selected, excluding two Chinese studies. The advanced search was refined to include only ‘article’ and ‘review’ publication types. Consequently, 142 non-relevant publications were excluded, such as Meeting Abstract (80), Correction (18), Letter (10), Early Access (9), Retracted Publication (7), Editorial Material (6), Book Chapters (4), and Proceeding Paper (3), Traction (3), Expression of Concern (1) and News Item (1). This final adjustment resulted in a total of 1,282 publications on celastrol being included.

### Data collection

To ensure data accuracy, the collection process was conducted in a single session (articles can be added retrospectively in WOS) (Lin et al. 2020). Data (record content = full record and cited references) were obtained from the WOS database as separate files with different types to meet the minimum criteria for each analysis software, Bibliometrix© (version 4.3.0) (Aria and Cuccurullo 2017) and CiteSpace© (version 6.3.R2) (Chen 2006), and VOSviewer© (version 1.6.19) (van Eck and Waltman 2010), resulting in three ‘plain text file’ types, save as ‘Download*.txt’ for 3 files. For each software type, combing undead files into a single file, ensuring no duplicates existed. Microsoft Excel was utilized to record publication information: number, year, country, journal, author, H-index, journal impact factor, and total times cited.

### Data analysis

For the analysis, we employed five distinct and innovative tools. GraphPad Prism 10 was utilized to delineate the frequency of publications, H-index, total times cited. SCImago Graphica was utilized to generate a global geographical map of publications. Subsequently, Bibliometrix (Aria and Cuccurullo 2017) was employed for various analyses, including the timeline of the top 10 authors by publication count, Sankey map of reference-author-keyword connections, word cloud of keywords, thematic map of authors and identify trends in the top keywords that rank for three time periods. Additionally, VOSviewer© (van Eck and Waltman 2010) was used to visualize and analyze the co-occurrence network among journals, authors, and keywords. To examine the national distribution of publications, national keywords, emerging terms, and keyword timelines, as well as to perform a journal dual-map overlay and subject cluster analysis, we conducted a comprehensive review of 1,282 records using CiteSpace (Chen 2006). This review spanned from January 1, 1997, to December 31, 2023, with a time slice set to one year and included centrality calculations.

## Results

### Overview of publications on celastrol

**Figure 1.**
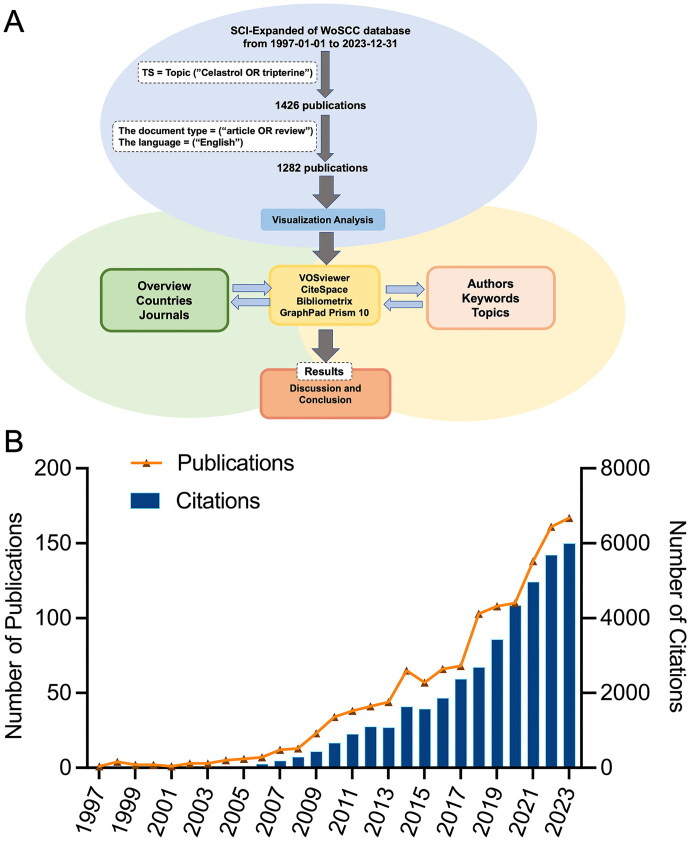
Study flowchart and celastrol publication overview. (A) Flowchart of the study methodology. (B) Trend chart depicting the annual publication count and citation metrics for celastrol research papers from 1997 to 2023.

According to the study’s flowchart (Figure 1(A)), the Web of Science (WoS) ultimately identified 1,282 publications, consisting solely of original research articles and review articles published in English, spanning the years 1997 to 2023. These papers amassed a total of 42,768 citations, averaging 33.36 citations per article.

The evolution of celastrol research can be divided into three phases based on annual publication outputs. Prior to 2008, celastrol research was relatively nascent, averaging about six papers per year. The total number of citations was 718. Between 2009 and 2017, there was a noticeable yearly increase in publications, averaging 48 publications per year with a growth rate of 5.6%. Over these nine years, the total number of citations amounted to 11,655. Since then, a more rapid growth in publications has begun, with an average of 157 publications per year and a growth rate reaching 12.8%. Citations during these six years surged to 27,131, nearly doubling the total from the previous nine years (Figure 1(B)).

### Geographic distribution and country analysis

**Figure 2.**
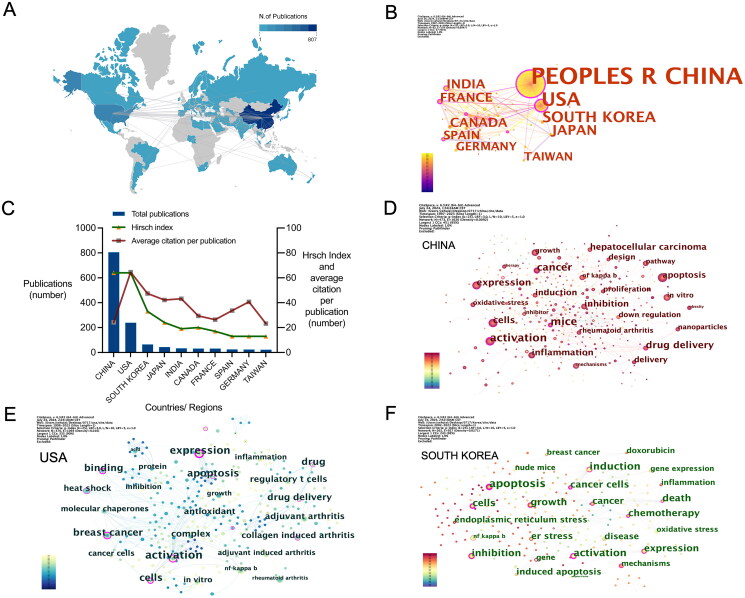
Geographic distribution and key countries/regions analysis in celastrol research. (A) Global distribution of celastrol research publications, with darker blue indicating higher publication volumes and gray representing the absence of publications. Gray lines illustrate inter-country connections. (B) Co-occurrence map highlighting the top 10 countries/regions based on publication volume. (C) Summary of publication volumes for the top 10 countries/regions. (D–F) Co-occurrence maps depicting the top 25 keywords in celastrol research papers from China (D) the United States. (E) and South Korea. (F) The size of each circle reflects the centrality value of the respective keyword, with purple circles indicating a centrality value of ≥0.1.

We examined the global distribution and principal countries/regions involved in celastrol research. Research on celastrol is being conducted across the globe, predominantly in Asia, North America, Europe, and Australia (Figure 2(A)). The top 10 countries/regions by publication are China, United States, South Korea, Japan, India, Canada, France, Spain, Germany and Taiwan (Figure 2(B), Table S1). In terms of the average number of citations per paper, the United States had the highest average citations at 64.53 times per paper, followed by South Korea (47.41 times) and India (43.14 times). The countries with the highest H-index values are China and the United States, both at 64 (Figure 2(C)).

To delve deeper into the primary research focuses on celastrol in the leading three countries (China, the USA, and South Korea), we conducted a co-occurrence analysis by extracting the top 25 most frequently occurring keywords from papers published in these countries. Prominent keywords such as ‘apoptosis,’ ‘expression,’ ‘activation,’ and ‘cells’ emerged with relatively high Betweenness centrality values. In China, notable keywords with a Betweenness centrality value of ≥0.1, excluding common ones, include ‘mice,’ ‘drug,’ and ‘delivery.’ In the USA, significant keywords are ‘breast cancer,’ ‘binding,’ ‘heat shock,’ ‘collagen-induced arthritis,’ ‘drug,’ and ‘molecular chaperones.’ In South Korea, important keywords are ‘induction,’ ‘death,’ ‘chemotherapy,’ ‘inhibition,’ ‘growth,’ and ‘mechanisms.’ (Figure 2(D–F), Tables 2)

### Journals and scientific disciplines

**Figure 3.**
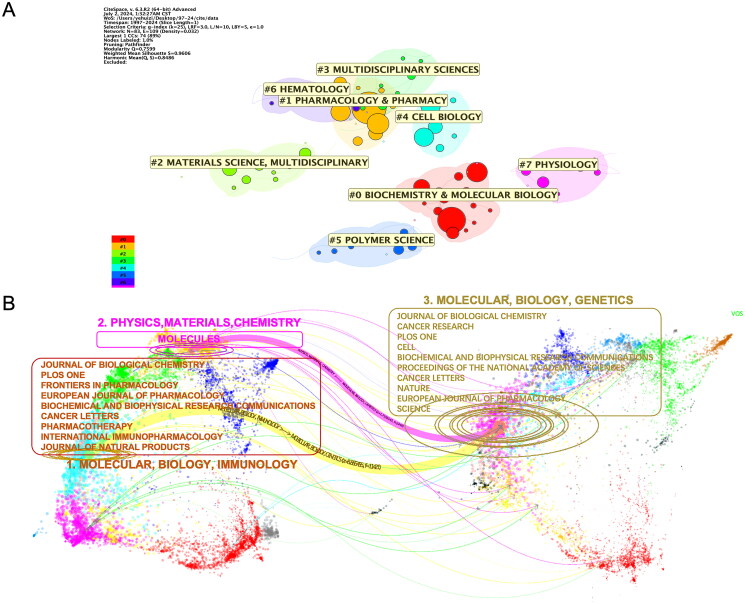
Journal analysis of celastrol research publications. (A) Cluster analysis of the primary subject categories within celastrol publications, conducted using CiteSpace. (B) Double overlay visualization of journals related to celastrol citations and citations, with cited journals represented on the left and citing journals on the right. The boxes highlight the top 10 cited/citing journals along with their corresponding subject classifications.

The analysis of the 1,282 publications on celastrol research, sourced from our study, indicates their publication across 536 peer-reviewed journals. A cluster analysis of the journals’ disciplines indicates that the top three categories are *Biochemistry* & *Molecular Biology*, *Pharmacology* & *Pharmacy* and *Materials Science, Multidisciplinary* (Figure 3(A)). Nearly, half of the top 10 journals are pharmacology, with the rest being comprehensive journals, and most of these journals have an impact factor (IF) around 5. *Frontiers in Pharmacology* stands out as the leading publication venue for celastrol research, contributing 36 publications, which is 2.81% of the total articles (Table 1). A dual-map overlay was employed for visualization, depicting the landscape of journals involved in celastrol research. In the visualization, journals citing celastrol research are shown on the left, while journals where celastrol research is cited are on the right. This analysis highlights two primary categories of citing journals, with the majority classified under MOLECULAR, BIOLOGY, IMMUNOLOGY, while a smaller portion falls under PHYSICS, MATERIALS, CHEMISTRY. Most of the cited journals are categorized under MOLECULAR, BIOLOGY, GENETICS (Figure 3(B)). Among the citing journals, two stand out with over 1,000 citations, *Journal of Biological Chemistry* and *Plos One*, with 1,040 and 1,025 citations. Within the top 10 co-cited journals, four have been cited more than 1,000 times, indicating their significant influence in the field. The *Journal of Biological Chemistry* leads with 1,543 co-citations, followed by *Cancer Research* with 1,061 co-citations, *Plos One* with 1060 co-citations, and *Cell* with 1007 co-citation (Table 2).

**Table 1.** The top 10 most productive journals related to celastrol.

Rank	Journal	Counts	Percentage	IF
1	*Frontiers in Pharmacology*	36	2.81	5.6
2	*PLos One*	28	2.18	3.7
3	*Molecules*	26	2.03	4.6
4	*Biomedicine Pharmacotherapy*	22	1.72	7.5
5	*European Journal of Pharmacology*	20	1.56	5.0
6	*International Immunopharmacology*	20	1.56	5.6
7	*Biochemical and Biophysical Research Communications*	19	1.48	3.1
8	*Pharmacotherapy*	19	1.48	4.1
9	*International Journal of Molecular Sciences*	18	1.40	5.6
10	*European Journal of Medicinal Chemistry*	17	1.33	6.7

IF: Impact factor issued in June 2023.

**Table 2.** The top 10 citing and cited journals related to celastrol.

Rank	Citing Journals (Left)		Cited Journals (Right)	
	Journal	Citations	Journal	Citations
1	*Journal of Biological Chemistry*	1040	*Journal of Biological Chemistry*	1543
2	*Plos One*	1025	*Cancer Research*	1061
3	*Frontiers in Pharmacology*	957	*Plos One*	1060
4	*European Journal of Pharmacology*	937	*Cell*	1007
5	*Biochemical and Biophysical Research Communications*	893	*Biochemical And Biophysical Research Communications*	830
6	*Cancer Letters*	891	*Proceedings of the National Academy of Sciences*	791
7	*Pharmacotherapy*	555	*Cancer Letters*	787
8	*International Immunopharmacology*	542	*Nature*	708
9	*Journal of Natural Products*	525	*European Journal of Pharmacology*	635
10	*Molecules*	500	*Science*	564

### Main authors

**Figure 4.**
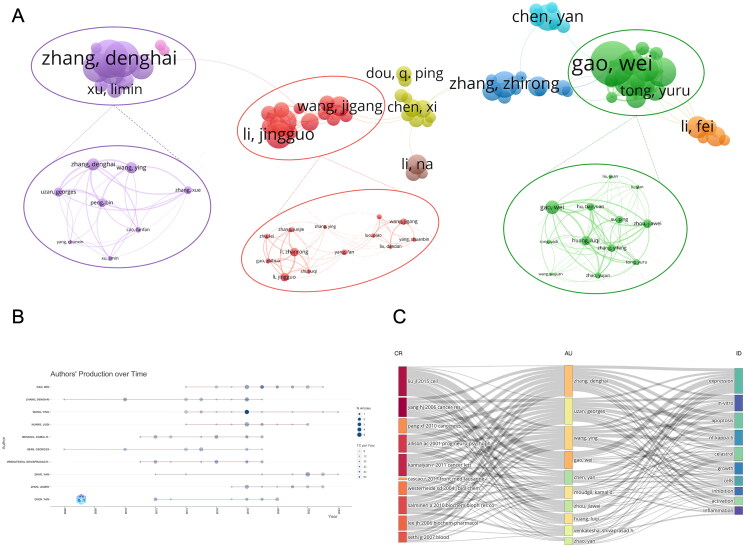
Analysis of authors in celastrol research. (A) Author co-occurrence map generated using VOSviewer. (B) Temporal distribution of publication status for the top 10 authors, with ‘N. article’ representing the number of articles and ‘TC per year’ denoting ‘Total Citations per Year.’ (C) Sankey diagram depicting the relationships among cited references, authors, and keywords. The left side illustrates the top 10 cited references, the center presents the top 10 authors ranked by publication count, and the right side displays the top 10 most frequently reported Keywords plus.

The dataset consists of 1,282 publications authored by a total of 7,107 individuals. A threshold criterion of a minimum of five published papers was established, leading to the manual consolidation of identical authors and resulting in 116 distinct author nodes. This process facilitated the construction of an author collaboration network comprising 70 nodes, which were categorized into nine distinct clusters. Within this network, three prominent collaborative clusters were identified: Wei Gao’s team from Capital Medical University, contributing 121 publications; Jingguo Li’s team from Zhengzhou University, with 98 publications; and Denghai Zhang’s team from Gongli Hospital of Shanghai Pudong New Area, which produced 95 publications (Figure 4(A)). Each of these teams comprises approximately ten members. In terms of total publications, the ranking is as follows: Wei Gao, Jingguo Li, and Denghai Zhang. When evaluated based on the average number of citations per paper, Jingguo Li’s team leads with an average of 27.65 citations per paper, followed closely by Denghai Zhang’s team with 27.25 citations, and Wei Gao’s team with 21.49 citations (Table S3). The publication timeline of the top ten authors indicates that Denghai Zhang’s team primarily concentrated its publications between 2006 and 2019, whereas Wei Gao’s team focused its publications from 2014 to 2022 (Figure 4(B)).

Figure 4(C) presents a Sankey diagram linking cited references, authors, and keywords. The left side lists the top 10 most cited celastrol research papers, the middle section shows the top 10 authors by publication count, and the right side highlights the top 10 relevant keywords. The gray lines between the three sections reflect the connections between each module. Detailed data can be found in Table S4 of the supplementary material. Wei Gao were most closely related to the 2015 article by JL Liu, particularly in terms of the keyword plus ‘gene expression.’ Denghai Zhang’s team (including members like Ying Wang and Uzan Georges) has strong connections to these top 10 publications and keywords, especially ‘apoptosis’ and ‘nf-kappa-b.’

### Core authors research keyword analysis

**Figure 5.**
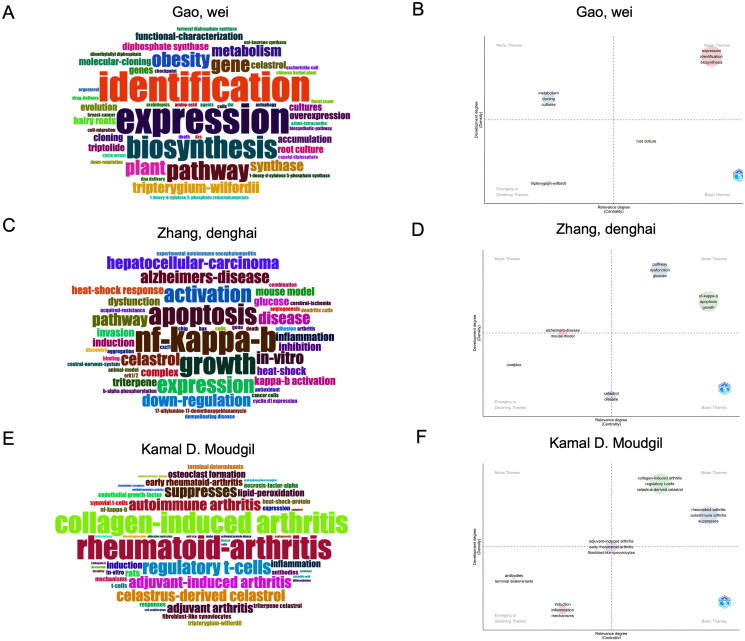
Keyword analysis of the top three celastrol researchers. (A) Word cloud representing the frequently utilized keywords by author Wei Gao. (B) Thematic map delineating the research areas explored by Wei Gao. (C) Word cloud illustrating keyword frequency in the studies conducted by Denghai Zhang. (D) Thematic map highlighting the research areas pursued by Denghai Zhang. (E) Word cloud reflecting the keywords employed by Kamal D. Moudgil. (F) Thematic overview map corresponding to the research conducted by Kamal D. Moudgil.

Among the top 10 authors in celastrol research, prominent figures include Wei Gao’s team, Denghai Zhang’s team, and Professor Kamal D. Moudgil. We visualized the frequency of keywords and described the thematic map for the top three authors by publication volume in celastrol research (Figure 5). In a word cloud, the size of a word indicates its frequency. The thematic map is divided into four quadrants based on centrality and density: the ‘top left’ quadrant represents niche themes, the ‘bottom left’ quadrant includes emerging or declining themes, the ‘upper right’ quadrant shows motor themes, and the ‘bottom right’ quadrant features basic themes (Hirsch 2005).

For Professor Wei Gao and his team, the high-frequency words in their word cloud are ‘identification,’ ‘expression,’ and ‘biosynthesis,’ which also appear in the upper right quadrant of their thematic map (Figure 5(A,B)).

In the case of Professor Denghai Zhang and his team, the most frequent words are ‘nf-kappa-b,’ ‘apoptosis,’ and ‘growth.’ Additional high-frequency terms such as ‘pathway,’ ‘dysfunction,’ and ‘glucose’ are also found in the upper right quadrant, forming two distinct clusters (Figure 5(C,D)).

Professor Kamal D. Moudgil’s key themes include ‘collagen-induced arthritis,’ ‘rheumatoid arthritis,’ and ‘regulatory T-cells.’ These terms, along with ‘autoimmune arthritis’ and ‘suppresses,’ are located in the upper right quadrant of the thematic map, indicating their importance and development within the field.

### Hotspots and frontiers

**Figure 6.**
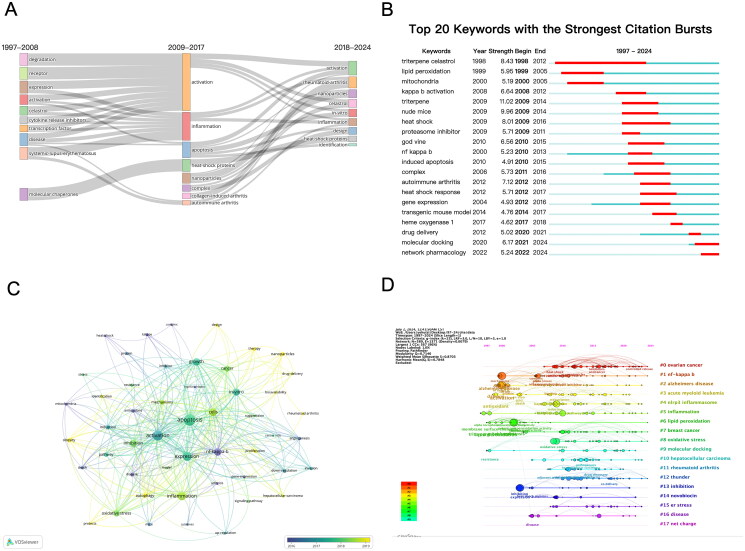
Trends in keywords and topics in celastrol research. (A) Sankey diagram illustrating the evolution of keywords in celastrol research across three distinct time periods: 1997-2008, 2009-2017, and 2018-2024. (B) Emergent analysis of the top 20 keywords identified from 1997 to 2023. (C) High-frequency keyword overlay map for celastrol (top 50 keywords), highlighting the primary years of occurrence for each keyword between 2016 and 2019. (D) Timeline map depicting the progression of keywords in celastrol research.

The analysis of keyword clouds effectively highlights the research hotspots and frontiers of celastrol (Figure 6(A)). From 1997 to 2008, studies primarily focused on ‘degradation,’ ‘receptor,’ ‘expression,’ ‘systemic-lupus-erythematosus,’ and ‘molecular chaperones.’ The period from 2009 to 2017 saw a shift towards ‘activation,’ ‘inflammation,’ and ‘apoptosis.’ Between 2018 and 2024, research interest pivoted to the therapeutic potential of diseases and modes of administration, with keywords such as ‘nanoparticles,’ ‘*in vitro*,’ and ‘design.’

Next, we analysed the emergence of the top 20 significant keywords in celastrol research, sorting them by their first occurrence to understand the evolving hotspots and trends, and to predict future research directions (Figure 6(B)). Prior to 2008, keywords like ‘lipid peroxidation’ and ‘mitochondria’ indicated early research focused on antioxidants. From 2009 to 2017, keywords such as ‘nude mice,’ ‘heat shock,’ ‘proteasome inhibitor,’ and ‘nf-kappa-b’ became prominent, indicating a shift towards anti-tumour and anti-inflammatory pharmacological research. In the last six years, the focus has been on the biological research of celastrol in the contexts of drug delivery and big data, with terms like ‘drug delivery,’ ‘molecular docking,’ and ‘network pharmacology.’

Using VOSviewer software, we mapped the co-occurrence of the top 50 keywords (excluding celastrol and related terms, and merging synonyms), and assigned colors by year to reflect their development over time (Figure 6(C)). Key node keywords included ‘apoptosis,’ ‘activation,’ ‘expression,’ ‘inflammation,’ and ‘cell,’ appearing 288, 231, 190, 181, and 152 times, respectively (Table S5), predominantly from 2016 to 2019.

Figure 6(D) is a timeline visualization of keyword clustering, highlighting recent research hotspots such as ‘ovarian cancer,’ ‘nf-kappa-b,’ ‘Alzheimer’s disease,’ ‘acute myeloid leukaemia,’ ‘NLRP3 inflammasome,’ ‘inflammation,’ ‘lipid peroxidation,’ ‘breast cancer,’ ‘oxidative stress,’ and ‘molecular docking.’ These clusters indicate that current celastrol research focuses on anti-inflammatory, anticancer, and other related mechanisms.

### Three main research areas of celastrol

Celastrol exhibits various pharmacological effects, notably anti-inflammatory and anticancer activities. However, its substantial toxicity limits its clinical application. Hence, this section explores these three research areas in greater detail.

**Figure 7.**
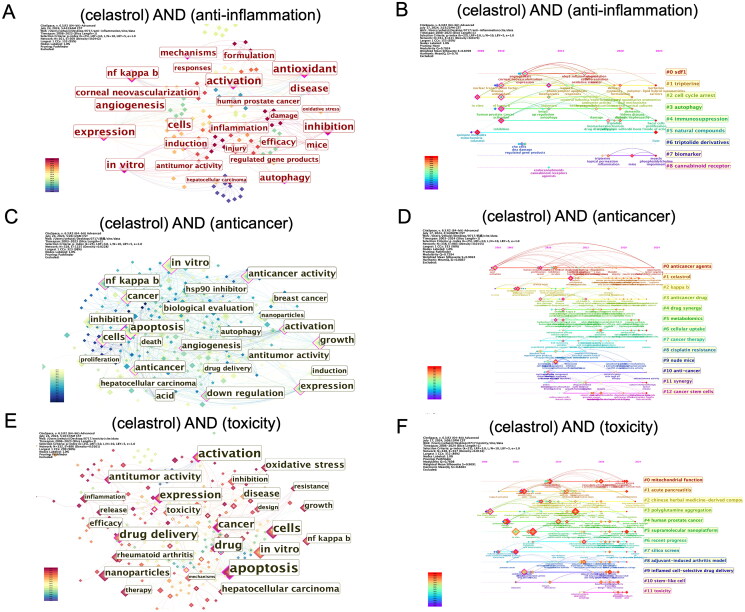
Analysis of the three major research areas in celastrol. (A) Co-occurrence map illustrating the top 25 keywords associated with celastrol and anti-inflammatory research. (B) Timeline depicting research trends focused on the anti-inflammatory properties of celastrol. (C) Co-occurrence map highlighting the top 25 keywords related to celastrol and anticancer research. (D) Timeline illustrating research developments centered on the anticancer properties of celastrol. (E) Co-occurrence map presenting the top 25 keywords pertinent to celastrol and toxicity studies. (F) Timeline showcasing research advancements related to the toxicity of celastrol.

By conducting a co-occurrence analysis of the keywords ‘celastrol’ and ‘anti-inflammation,’ we identified keywords with betweenness centrality ≥0.1, such as ‘antioxidant,’ ‘inhibition,’ ‘nf-kappa-b,’ ‘autophagy,’ and ‘angiogenesis’ (Figure 7(A)). Figure 7(B) outlines nine major research clusters over time, focusing on themes like ‘sdf1,’ ‘cell cycle arrest,’ ‘autophagy,’ ‘immunosuppression,’ and ‘natural compounds.’

Similarly, the co-occurrence analysis of ‘celastrol’ and ‘anticancer’ revealed high-centrality keywords such as ‘apoptosis,’ ‘nf-kappa-b,’ ‘growth,’ ‘angiogenesis,’ and ‘down regulation’ (Figure 7(C)). Figure 7(D) displays thirteen main research clusters over time, covering topics like ‘anticancer drug,’ ‘drug synergy,’ ‘metabolomics,’ and ‘synergy.’

Lastly, examining the co-occurrence of ‘celastrol’ and ‘toxicity’ revealed keywords with centrality ≥0.1, like ‘apoptosis,’ ‘drug delivery,’ ‘antitumor activity,’ ‘drug,’ and ‘*in vitro*’ (Figure 7(E)). Figure 7(F) identifies twelve primary research clusters, including ‘mitochondrial function,’ ‘acute pancreatitis,’ ‘polyglutamine aggregation,’ ‘supramolecular nanoplatform,’ ‘silico screen,’ and ‘inflamed cell-selective drug delivery.’ [Insert Figure 7 near here]

## Discussion

Research on celastrol has continued for decades due to its varied pharmacological activities, establishing it as a highly promising lead compound (Corson and Crews 2007). This study collected celastrol related literature from January 1997 to December 2023. Utilizing bibliometric methods, we visually analyzed publication volume, countries/regions, authors, keywords and disciplinary fields. The findings revealed temporal shifts in celastrol research, with China, the USA, and South Korea as the main contributors, each focusing on distinct aspects. Several major research teams have emerged, each with specific focal points. Additionally, highly cited papers indicate shifts in research emphasis. Literature analysis also suggests future research directions.

Research intensity and depth in the studies of celastrol have demonstrated a significant increase over time. Since 1997, both the quantity and scope of publications related to celastrol have markedly risen, particularly in the last six years, indicating heightened research activity and impact. This trend suggests that celastrol, known for its diverse pharmacological properties, is attracting considerable attention from the scientific community. Furthermore, we have noted that research hotspots and priorities have evolved across different time periods. Early studies focused on observing pharmacological effects (Allison et al. 2001), while later research shifted towards discovering molecular targets. For example, ‘proteasome inhibitor’ and ‘nf-kappa-b’ emerged as key keywords from 2009 to 2017 (Freudlsperger et al. 2013; Boridy et al. 2014). Recent studies, from 2017 onwards, have delved into pharmacological mechanisms at a more detailed sub-molecular level and through complex systems biology approaches, with keywords like ‘molecular docking’ and ‘network pharmacology’ becoming prominent (Liang et al. 2023).

The analysis also uncovered variations in the primary countries involved in celastrol research and their specific areas of interest. The indicator of "producing countries" highlights significant differences in research focus and innovation. Analysing outputs by country reveals regional strengths and priorities, offering insights for global collaboration and resource allocation. China has the highest publication volume in this field, which can be attributed to its extensive history of traditional herbal medicine use (Tang et al. 2008). This is followed by the United States and South Korea. Notably, although the number of papers published in the United States is only one-third of that in China, the average number of citations per paper in the USA is 2.6 multiple times higher, and the H-index is comparable to that of China. This suggests that the quality of the USA publications is higher, and celastrol research from the USA has a greater impact.

From the national keyword co-occurrence map of the top three publishing countries, it is evident that research in China primarily focuses on the basic pharmacology of anticancer effects both *in vitro* and *in vivo*, particularly concerning liver cancer (Zhou et al. 2023), and on novel drug delivery systems (Yu et al. 2022). According to the latest data from the WHO, released on February 1, 2024, in the report ‘Global Cancer Burden Growing, Amidst Mounting Need for Services,’ liver cancer incidence has risen to fourth place in China, with mortality rates still the second highest (Organization WH 2024). The numerous studies on new drug delivery systems may be due to Chinese researchers’ awareness of celastrol toxicity. Clinical practice in China already includes various celastrol preparations (where celastrol is the main active ingredient), and these can cause toxic reactions in some patients (Guo et al. 2023). Therefore, the development of new drug delivery systems aimed at reducing toxicity while maintaining efficacy is receiving increasing attention. In the United States, research on breast cancer is significant (Dandawate et al. 2016), likely driven by the rising incidence of breast cancer in the country. By 2022, breast cancer deaths had climbed to the second highest cause of cancer mortality (Giaquinto et al. 2022). Research in South Korea focuses primarily on cancer treatment, particularly the mechanisms of action of chemotherapy drugs (Soe et al. 2018).

Analysis identified several prominent research teams with distinct focal areas. Cluster analysis of publication volume revealed three core teams: Gao Wei from Capital Medical University, Jingguo Li from Zhengzhou University, and Denghai Zhang from Gongli Hospital of Shanghai Pudong New Area, all based in China. Gao Wei’s team investigates celastrol biosynthesis (Zhou et al. 2019; Luo et al. 2023), Jingguo Li’s team develops nanomedicine materials incorporating celastrol (Li et al. 2015; An et al. 2020), and Denghai Zhang’s team explores mechanisms for treating chronic diseases such as cancer, Alzheimer’s (Cao et al. 2018), and insulin resistance (Zhang et al. 2018). Despite being located in the same country, these teams exhibit minimal collaboration and communication. Enhancing cooperation could expedite advancements in celastrol research. Beyond China, Professor KD Moudgil’s team at the University of Maryland School of Medicine, although not a distinct cluster in the analysis, ranks among the top 10 globally for celastrol publications. Their research centres on using celastrol to treat autoimmune inflammatory diseases.

Literature analysis identifies emerging research directions, with highly cited papers reflecting shifting priorities. Key publications on celastrol include Yang et al. (2006) in *Cancer Research* (Yang et al. 2006), Liu et al. (2015) in *Cell* (Liu et al. 2015) and Kannaiyan et al. (2011) in *Cancer Letters* (Kannaiyan et al. 2011). Yang et al. demonstrated the efficacy of celastrol as a potent proteasome inhibitor for cancer treatment, generating significant interest. Liu et al.’s discovery of celastrol’s anti-obesity effects expanded its applications to metabolic diseases. Additionally, Kannaiyan et al.’s comprehensive review has been influential, highlighting broad interest in celastrol’s potential. These trends indicate a shift in celastrol research from anticancer applications to metabolic disease interventions.

Analysing the three sub-fields of celastrol research – anti-inflammatory, anti-tumor, and toxicity – highlights key areas of interest. In anti-inflammatory research, ‘nf-kappa-b,’ ‘angiogenesis,’ ‘autophagy,’ and ‘antioxidant’ are major focus points (Allison et al. 2001; Kim et al. 2013; Li et al. 2020; Yang et al. 2022). In anti-tumour studies, researchers concentrate on ‘apoptosis,’ ‘down regulation,’ ‘nf-kappa-b,’ and ‘angiogenesis’ (Deng et al. 2021; Youns et al. 2021; Lai et al. 2024). Both fields emphasize nf-kappa-b and angiogenesis due to their critical roles in inflammation and cancer development, with celastrol impacting these processes. In anticancer research, ‘down regulation’ indicates celastrol inhibitory effects, such as on nf-kappa-b (Sethi et al. 2007; Shen et al. 2016), HSP90 (Zhang et al. 2009; Peng et al. 2010), and the proteasome (Yang et al. 2006; Zhong et al. 2019). Toxicity research focuses on structural modification (Xu et al. 2023), drug combination (Liu et al. 2021), and drug delivery (Li et al. 2024).

### Strengths and limitations

This study is strengthened by its application of five bibliometric tools to comprehensively analyze celastrol-related English publications over the past 27 years, enabling a detailed assessment of disparities across countries, leading research teams, and fields. This provides valuable insights for emerging researchers investigating new directions. However, limitations exist due to the dynamic nature of bibliometric data, which may change over time. Our analysis focused solely on English-language papers published within a specific timeframe, omitting older literature, recent publications, and other publication types such as conference papers and letters. Furthermore, the reliance on the H-index as a measure of publication quality may not fully reflect the impact of high-quality papers or preliminary studies that lack extensive citations. Additionally, celastrol is only one active monomer in clinical preparations, and our study did not address other components such as Triptolide or Wilformine.

## Conclusions

Research papers on celastrol have demonstrated an increasing trend in both intensity and depth over time and can be categorized into three stages, each with a shifting focus. The leading countries in terms of publication volume are China, the United States, and South Korea, each exhibiting distinct research interests, with several prominent research teams focusing on various aspects of celastrol. Among the three primary research areas—anti-inflammatory, antitumor, and toxicity—the first two show considerable overlap, while recent studies have increasingly emphasized the toxic effects of celastrol and strategies to mitigate them. This study adopts a bibliometric approach to systematically analyze global research trends, key contributors, and thematic focuses related to celastrol. The objective is to map the global research landscape, including publication trends, country-specific outputs, and collaborative networks, identify research hotspots and major thematic areas, and uncover potential gaps and future research directions. By providing comprehensive insights and actionable guidance, this study aims to inspire further investigations into the multi-target effects of celastrol, particularly through advanced methodologies like network biology and molecular simulation.

## Supplementary Material

Supplementary materials.pptx

## Data Availability

All data supporting the findings of this study are included within the main text of the article and its Supplementary Information files.
