# Supplementary material for: Characterization of global research trends and prospects on celastrol, a principal bioactive ingredient of Tripterygium wilfordii Hook F: bibliometric analysis
Source: Pharm Biol. 2025 Jan 2;63(1):15–26. doi: 10.1080/13880209.2024.2443424 (PMC11703041; doi:10.1080/13880209.2024.2443424)
Supplement: Supplementary materials.pptx [file IPHB_A_2443424_SM5499.pptx]

## Slide 1
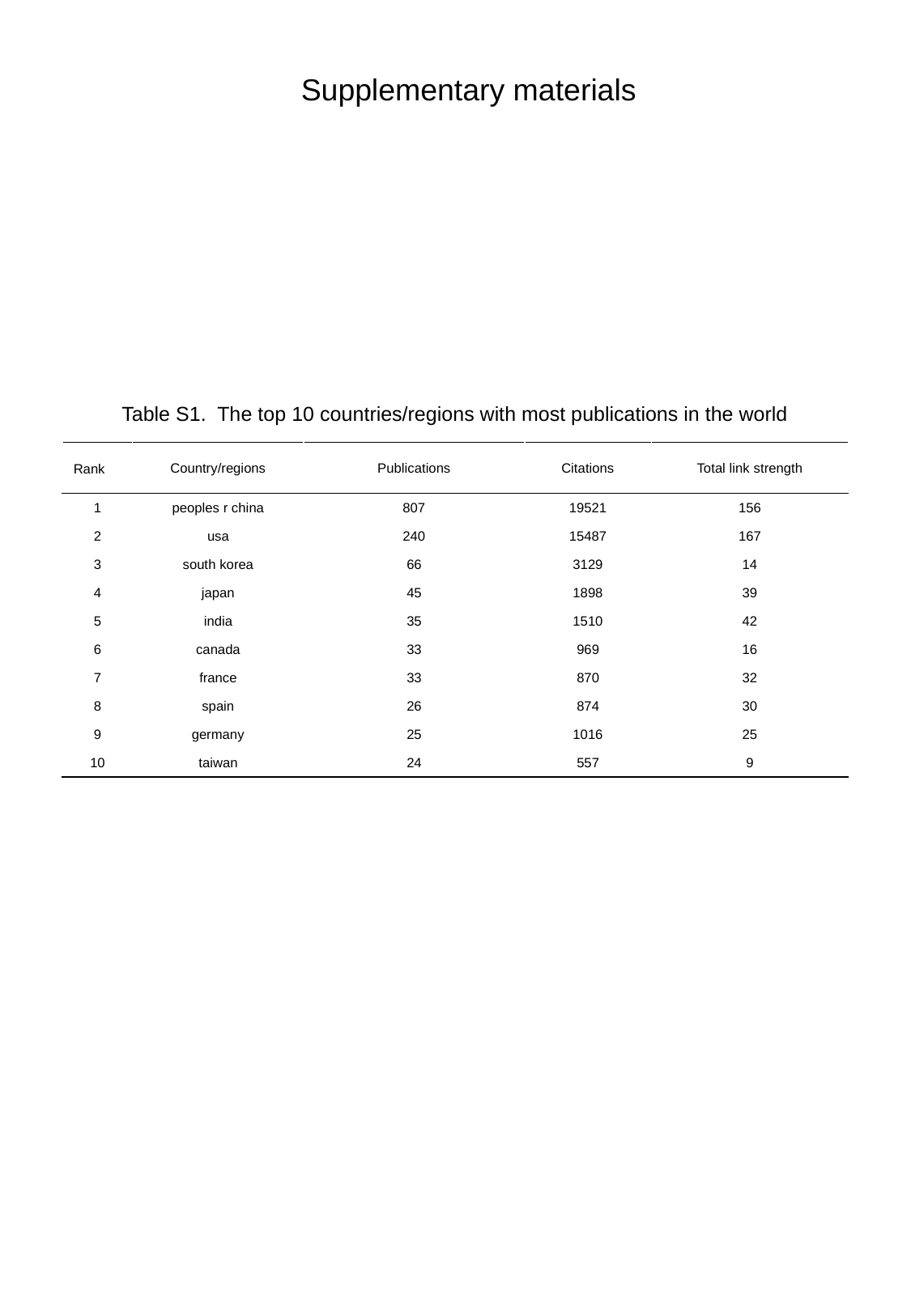

Supplementary materials
Table S1. The top 10 countries/regions with most publications in the world
| Rank | Country/regions | Publications | Citations | Total link strength |
| --- | --- | --- | --- | --- |
| 1 | peoples r china | 807 | 19521 | 156 |
| 2 | usa | 240 | 15487 | 167 |
| 3 | south korea | 66 | 3129 | 14 |
| 4 | japan | 45 | 1898 | 39 |
| 5 | india | 35 | 1510 | 42 |
| 6 | canada | 33 | 969 | 16 |
| 7 | france | 33 | 870 | 32 |
| 8 | spain | 26 | 874 | 30 |
| 9 | germany | 25 | 1016 | 25 |
| 10 | taiwan | 24 | 557 | 9 |

## Slide 2
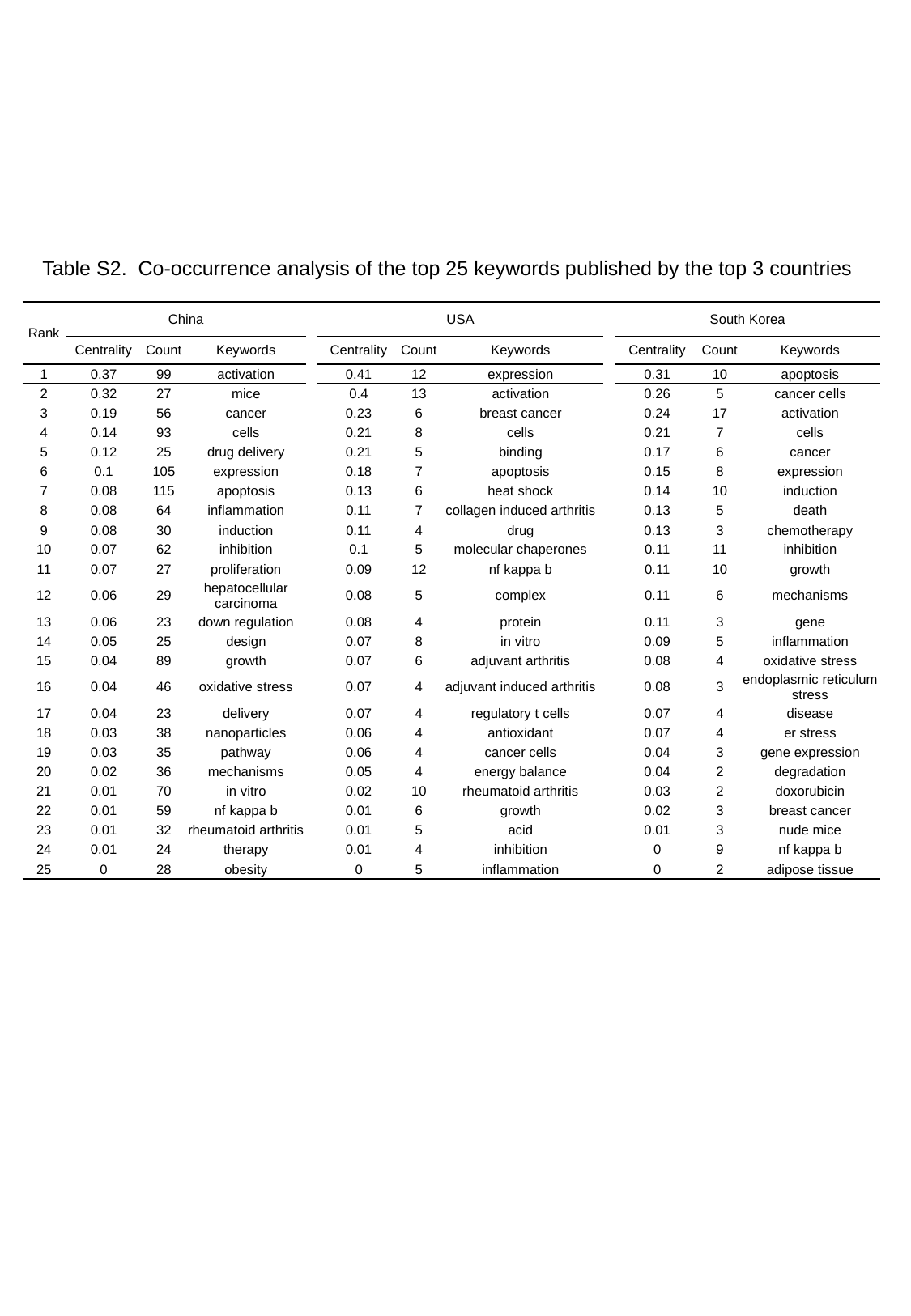

Table S2. Co-occurrence analysis of the top 25 keywords published by the top 3 countries
| Rank | China | | | | USA | | | | South Korea | | |
| --- | --- | --- | --- | --- | --- | --- | --- | --- | --- | --- | --- |
| | Centrality | Count | Keywords | | Centrality | Count | Keywords | | Centrality | Count | Keywords |
| 1 | 0.37 | 99 | activation | | 0.41 | 12 | expression | | 0.31 | 10 | apoptosis |
| 2 | 0.32 | 27 | mice | | 0.4 | 13 | activation | | 0.26 | 5 | cancer cells |
| 3 | 0.19 | 56 | cancer | | 0.23 | 6 | breast cancer | | 0.24 | 17 | activation |
| 4 | 0.14 | 93 | cells | | 0.21 | 8 | cells | | 0.21 | 7 | cells |
| 5 | 0.12 | 25 | drug delivery | | 0.21 | 5 | binding | | 0.17 | 6 | cancer |
| 6 | 0.1 | 105 | expression | | 0.18 | 7 | apoptosis | | 0.15 | 8 | expression |
| 7 | 0.08 | 115 | apoptosis | | 0.13 | 6 | heat shock | | 0.14 | 10 | induction |
| 8 | 0.08 | 64 | inflammation | | 0.11 | 7 | collagen induced arthritis | | 0.13 | 5 | death |
| 9 | 0.08 | 30 | induction | | 0.11 | 4 | drug | | 0.13 | 3 | chemotherapy |
| 10 | 0.07 | 62 | inhibition | | 0.1 | 5 | molecular chaperones | | 0.11 | 11 | inhibition |
| 11 | 0.07 | 27 | proliferation | | 0.09 | 12 | nf kappa b | | 0.11 | 10 | growth |
| 12 | 0.06 | 29 | hepatocellular carcinoma | | 0.08 | 5 | complex | | 0.11 | 6 | mechanisms |
| 13 | 0.06 | 23 | down regulation | | 0.08 | 4 | protein | | 0.11 | 3 | gene |
| 14 | 0.05 | 25 | design | | 0.07 | 8 | in vitro | | 0.09 | 5 | inflammation |
| 15 | 0.04 | 89 | growth | | 0.07 | 6 | adjuvant arthritis | | 0.08 | 4 | oxidative stress |
| 16 | 0.04 | 46 | oxidative stress | | 0.07 | 4 | adjuvant induced arthritis | | 0.08 | 3 | endoplasmic reticulum stress |
| 17 | 0.04 | 23 | delivery | | 0.07 | 4 | regulatory t cells | | 0.07 | 4 | disease |
| 18 | 0.03 | 38 | nanoparticles | | 0.06 | 4 | antioxidant | | 0.07 | 4 | er stress |
| 19 | 0.03 | 35 | pathway | | 0.06 | 4 | cancer cells | | 0.04 | 3 | gene expression |
| 20 | 0.02 | 36 | mechanisms | | 0.05 | 4 | energy balance | | 0.04 | 2 | degradation |
| 21 | 0.01 | 70 | in vitro | | 0.02 | 10 | rheumatoid arthritis | | 0.03 | 2 | doxorubicin |
| 22 | 0.01 | 59 | nf kappa b | | 0.01 | 6 | growth | | 0.02 | 3 | breast cancer |
| 23 | 0.01 | 32 | rheumatoid arthritis | | 0.01 | 5 | acid | | 0.01 | 3 | nude mice |
| 24 | 0.01 | 24 | therapy | | 0.01 | 4 | inhibition | | 0 | 9 | nf kappa b |
| 25 | 0 | 28 | obesity | | 0 | 5 | inflammation | | 0 | 2 | adipose tissue |

## Slide 3
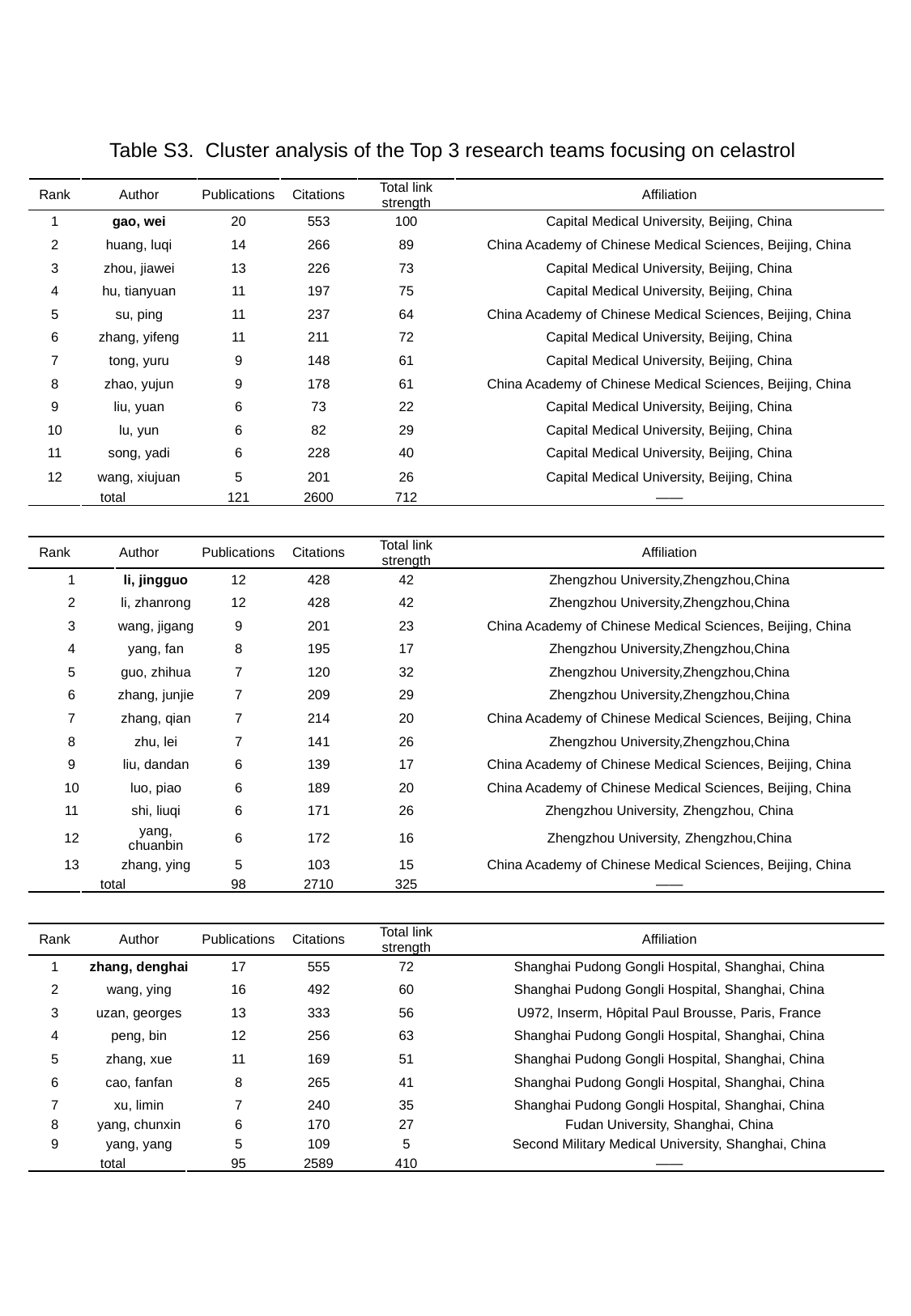

Table S3. Cluster analysis of the Top 3 research teams focusing on celastrol
| Rank | Author | author | author | Publications | Citations | Total link strength | Affiliation |
| --- | --- | --- | --- | --- | --- | --- | --- |
| 1 | gao, wei | gao, wei | gao, wei | 20 | 553 | 100 | Capital Medical University, Beijing, China |
| 2 | huang, luqi | huang, luqi | huang, luqi | 14 | 266 | 89 | China Academy of Chinese Medical Sciences, Beijing, China |
| 3 | zhou, jiawei | zhou, jiawei | zhou, jiawei | 13 | 226 | 73 | Capital Medical University, Beijing, China |
| 4 | hu, tianyuan | hu, tianyuan | hu, tianyuan | 11 | 197 | 75 | Capital Medical University, Beijing, China |
| 5 | su, ping | su, ping | su, ping | 11 | 237 | 64 | China Academy of Chinese Medical Sciences, Beijing, China |
| 6 | zhang, yifeng | zhang, yifeng | zhang, yifeng | 11 | 211 | 72 | Capital Medical University, Beijing, China |
| 7 | tong, yuru | tong, yuru | tong, yuru | 9 | 148 | 61 | Capital Medical University, Beijing, China |
| 8 | zhao, yujun | zhao, yujun | zhao, yujun | 9 | 178 | 61 | China Academy of Chinese Medical Sciences, Beijing, China |
| 9 | liu, yuan | liu, yuan | liu, yuan | 6 | 73 | 22 | Capital Medical University, Beijing, China |
| 10 | lu, yun | lu, yun | lu, yun | 6 | 82 | 29 | Capital Medical University, Beijing, China |
| 11 | song, yadi | song, yadi | song, yadi | 6 | 228 | 40 | Capital Medical University, Beijing, China |
| 12 | wang, xiujuan | wang, xiujuan | wang, xiujuan | 5 | 201 | 26 | Capital Medical University, Beijing, China |
| total | | | | 121 | 2600 | 712 | —— |
| | | | | | | | |
| | | | | | | | |
| Rank | Author | author | author | Publications | Citations | Total link strength | Affiliation |
| 1 | | li, jingguo | li, jingguo | 12 | 428 | 42 | Zhengzhou University,Zhengzhou,China |
| 2 | | li, zhanrong | li, zhanrong | 12 | 428 | 42 | Zhengzhou University,Zhengzhou,China |
| 3 | | wang, jigang | wang, jigang | 9 | 201 | 23 | China Academy of Chinese Medical Sciences, Beijing, China |
| 4 | | yang, fan | yang, fan | 8 | 195 | 17 | Zhengzhou University,Zhengzhou,China |
| 5 | | guo, zhihua | guo, zhihua | 7 | 120 | 32 | Zhengzhou University,Zhengzhou,China |
| 6 | | zhang, junjie | zhang, junjie | 7 | 209 | 29 | Zhengzhou University,Zhengzhou,China |
| 7 | | zhang, qian | zhang, qian | 7 | 214 | 20 | China Academy of Chinese Medical Sciences, Beijing, China |
| 8 | | zhu, lei | zhu, lei | 7 | 141 | 26 | Zhengzhou University,Zhengzhou,China |
| 9 | | liu, dandan | liu, dandan | 6 | 139 | 17 | China Academy of Chinese Medical Sciences, Beijing, China |
| 10 | | luo, piao | luo, piao | 6 | 189 | 20 | China Academy of Chinese Medical Sciences, Beijing, China |
| 11 | | shi, liuqi | shi, liuqi | 6 | 171 | 26 | Zhengzhou University, Zhengzhou, China |
| 12 | | yang, chuanbin | yang, chuanbin | 6 | 172 | 16 | Zhengzhou University, Zhengzhou,China |
| 13 | | zhang, ying | zhang, ying | 5 | 103 | 15 | China Academy of Chinese Medical Sciences, Beijing, China |
| total | | | | 98 | 2710 | 325 | —— |
| | | | | | | | |
| | | | | | | | |
| Rank | Author | author | author | Publications | Citations | Total link strength | Affiliation |
| 1 | zhang, denghai | zhang, denghai | zhang, denghai | 17 | 555 | 72 | Shanghai Pudong Gongli Hospital, Shanghai, China |
| 2 | wang, ying | wang, ying | wang, ying | 16 | 492 | 60 | Shanghai Pudong Gongli Hospital, Shanghai, China |
| 3 | uzan, georges | uzan, georges | uzan, georges | 13 | 333 | 56 | U972, Inserm, Hôpital Paul Brousse, Paris, France |
| 4 | peng, bin | peng, bin | peng, bin | 12 | 256 | 63 | Shanghai Pudong Gongli Hospital, Shanghai, China |
| 5 | zhang, xue | zhang, xue | zhang, xue | 11 | 169 | 51 | Shanghai Pudong Gongli Hospital, Shanghai, China |
| 6 | cao, fanfan | cao, fanfan | cao, fanfan | 8 | 265 | 41 | Shanghai Pudong Gongli Hospital, Shanghai, China |
| 7 | xu, limin | xu, limin | xu, limin | 7 | 240 | 35 | Shanghai Pudong Gongli Hospital, Shanghai, China |
| 8 | yang, chunxin | yang, chunxin | yang, chunxin | 6 | 170 | 27 | Fudan University, Shanghai, China |
| 9 | yang, yang | yang, yang | yang, yang | 5 | 109 | 5 | Second Military Medical University, Shanghai, China |
| total | | | | 95 | 2589 | 410 | —— |
| | | | | | | | |

## Slide 4
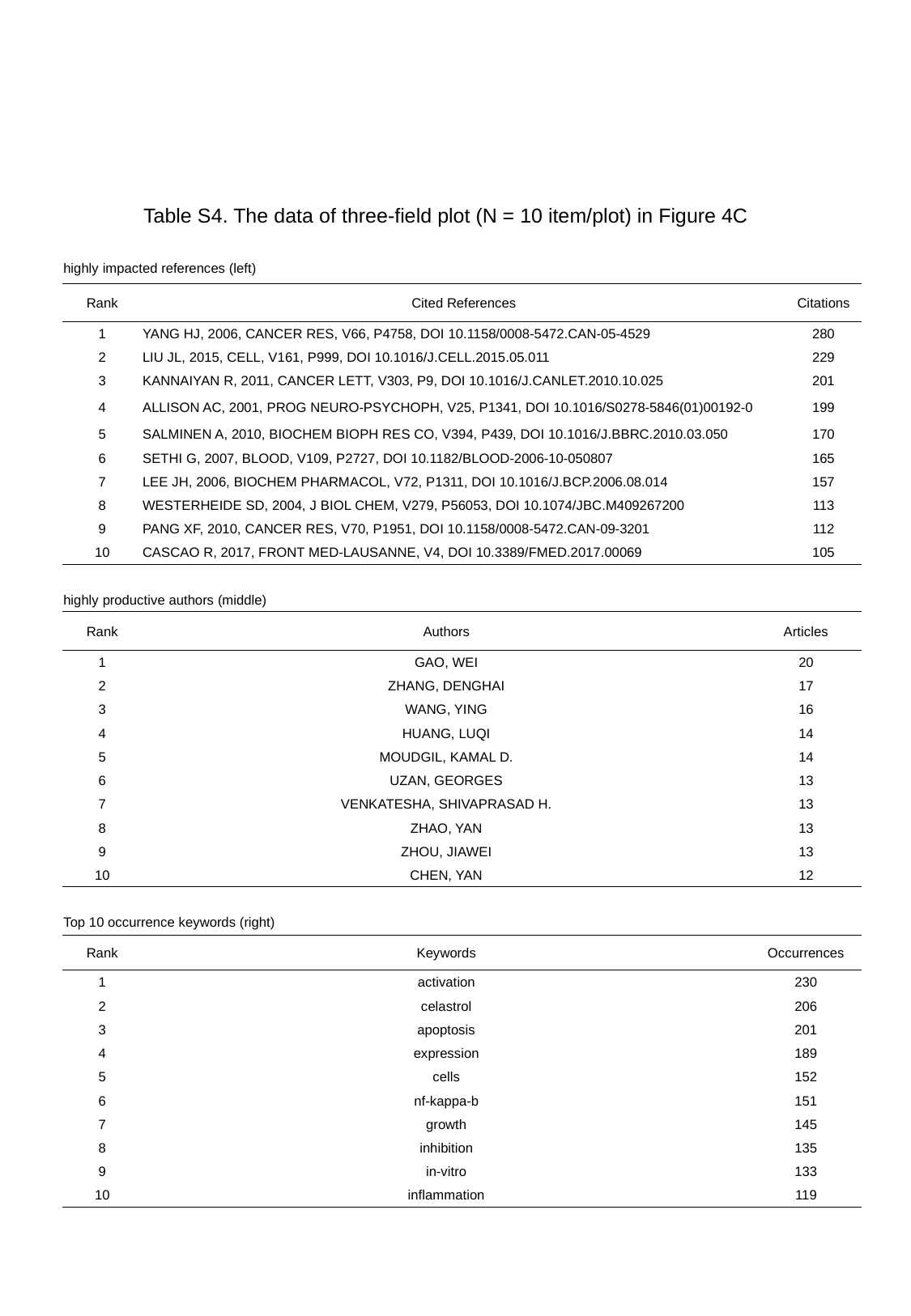

Table S4. The data of three-field plot (N = 10 item/plot) in Figure 4C
| highly impacted references (left) | | | |
| --- | --- | --- | --- |
| Rank | Cited References | | Citations |
| 1 | YANG HJ, 2006, CANCER RES, V66, P4758, DOI 10.1158/0008-5472.CAN-05-4529 | | 280 |
| 2 | LIU JL, 2015, CELL, V161, P999, DOI 10.1016/J.CELL.2015.05.011 | | 229 |
| 3 | KANNAIYAN R, 2011, CANCER LETT, V303, P9, DOI 10.1016/J.CANLET.2010.10.025 | | 201 |
| 4 | ALLISON AC, 2001, PROG NEURO-PSYCHOPH, V25, P1341, DOI 10.1016/S0278-5846(01)00192-0 | | 199 |
| 5 | SALMINEN A, 2010, BIOCHEM BIOPH RES CO, V394, P439, DOI 10.1016/J.BBRC.2010.03.050 | | 170 |
| 6 | SETHI G, 2007, BLOOD, V109, P2727, DOI 10.1182/BLOOD-2006-10-050807 | | 165 |
| 7 | LEE JH, 2006, BIOCHEM PHARMACOL, V72, P1311, DOI 10.1016/J.BCP.2006.08.014 | | 157 |
| 8 | WESTERHEIDE SD, 2004, J BIOL CHEM, V279, P56053, DOI 10.1074/JBC.M409267200 | | 113 |
| 9 | PANG XF, 2010, CANCER RES, V70, P1951, DOI 10.1158/0008-5472.CAN-09-3201 | | 112 |
| 10 | CASCAO R, 2017, FRONT MED-LAUSANNE, V4, DOI 10.3389/FMED.2017.00069 | | 105 |
| | | | |
| highly productive authors (middle) | | | |
| Rank | Authors | Articles | Articles |
| 1 | GAO, WEI | 20 | 20 |
| 2 | ZHANG, DENGHAI | 17 | 17 |
| 3 | WANG, YING | 16 | 16 |
| 4 | HUANG, LUQI | 14 | 14 |
| 5 | MOUDGIL, KAMAL D. | 14 | 14 |
| 6 | UZAN, GEORGES | 13 | 13 |
| 7 | VENKATESHA, SHIVAPRASAD H. | 13 | 13 |
| 8 | ZHAO, YAN | 13 | 13 |
| 9 | ZHOU, JIAWEI | 13 | 13 |
| 10 | CHEN, YAN | 12 | 12 |
| | | | |
| Top 10 occurrence keywords (right) | | | |
| Rank | Keywords | Occurrences | Occurrences |
| 1 | activation | 230 | 230 |
| 2 | celastrol | 206 | 206 |
| 3 | apoptosis | 201 | 201 |
| 4 | expression | 189 | 189 |
| 5 | cells | 152 | 152 |
| 6 | nf-kappa-b | 151 | 151 |
| 7 | growth | 145 | 145 |
| 8 | inhibition | 135 | 135 |
| 9 | in-vitro | 133 | 133 |
| 10 | inflammation | 119 | 119 |

## Slide 5
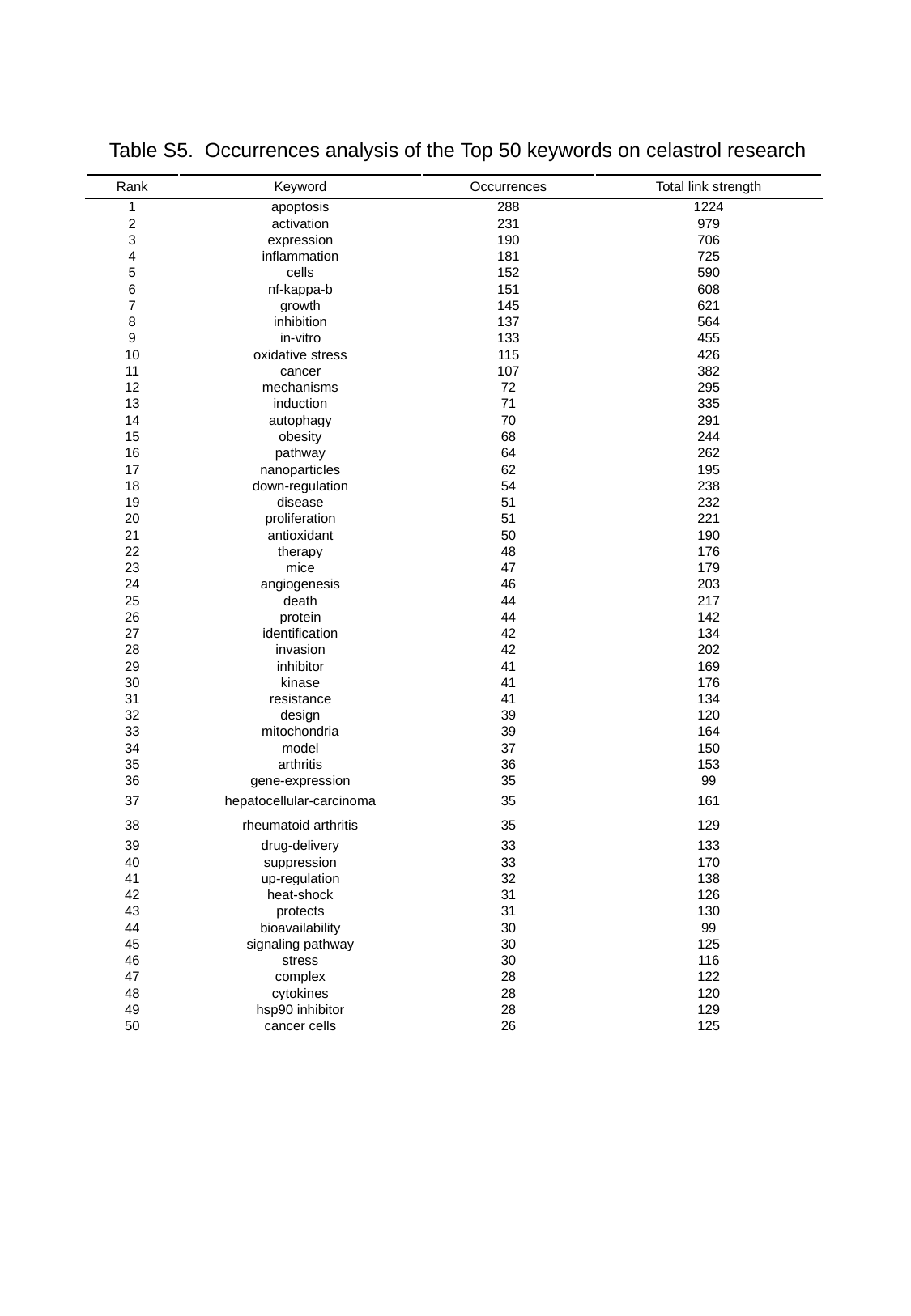

Table S5. Occurrences analysis of the Top 50 keywords on celastrol research
| Rank | Keyword | Occurrences | Total link strength |
| --- | --- | --- | --- |
| 1 | apoptosis | 288 | 1224 |
| 2 | activation | 231 | 979 |
| 3 | expression | 190 | 706 |
| 4 | inflammation | 181 | 725 |
| 5 | cells | 152 | 590 |
| 6 | nf-kappa-b | 151 | 608 |
| 7 | growth | 145 | 621 |
| 8 | inhibition | 137 | 564 |
| 9 | in-vitro | 133 | 455 |
| 10 | oxidative stress | 115 | 426 |
| 11 | cancer | 107 | 382 |
| 12 | mechanisms | 72 | 295 |
| 13 | induction | 71 | 335 |
| 14 | autophagy | 70 | 291 |
| 15 | obesity | 68 | 244 |
| 16 | pathway | 64 | 262 |
| 17 | nanoparticles | 62 | 195 |
| 18 | down-regulation | 54 | 238 |
| 19 | disease | 51 | 232 |
| 20 | proliferation | 51 | 221 |
| 21 | antioxidant | 50 | 190 |
| 22 | therapy | 48 | 176 |
| 23 | mice | 47 | 179 |
| 24 | angiogenesis | 46 | 203 |
| 25 | death | 44 | 217 |
| 26 | protein | 44 | 142 |
| 27 | identification | 42 | 134 |
| 28 | invasion | 42 | 202 |
| 29 | inhibitor | 41 | 169 |
| 30 | kinase | 41 | 176 |
| 31 | resistance | 41 | 134 |
| 32 | design | 39 | 120 |
| 33 | mitochondria | 39 | 164 |
| 34 | model | 37 | 150 |
| 35 | arthritis | 36 | 153 |
| 36 | gene-expression | 35 | 99 |
| 37 | hepatocellular-carcinoma | 35 | 161 |
| 38 | rheumatoid arthritis | 35 | 129 |
| 39 | drug-delivery | 33 | 133 |
| 40 | suppression | 33 | 170 |
| 41 | up-regulation | 32 | 138 |
| 42 | heat-shock | 31 | 126 |
| 43 | protects | 31 | 130 |
| 44 | bioavailability | 30 | 99 |
| 45 | signaling pathway | 30 | 125 |
| 46 | stress | 30 | 116 |
| 47 | complex | 28 | 122 |
| 48 | cytokines | 28 | 120 |
| 49 | hsp90 inhibitor | 28 | 129 |
| 50 | cancer cells | 26 | 125 |
